# *Plasmodium vivax dhfr *and *dhps *mutations in isolates from Madagascar and therapeutic response to sulphadoxine-pyrimethamine

**DOI:** 10.1186/1475-2875-7-35

**Published:** 2008-02-26

**Authors:** Céline Barnadas, Magali Tichit, Christiane Bouchier, Arsène Ratsimbasoa, Laurence Randrianasolo, Rogelin Raherinjafy, Martial Jahevitra, Stéphane Picot, Didier Ménard

**Affiliations:** 1 EA4170, Parasitology and Tropical Medicine, University Lyon 1, Lyon, France; 2 Epidemiology Unit, Institut Pasteur de Madagascar, Antananarivo, Madagascar; 3 Malaria Unit Research, Institut Pasteur de Madagascar, Antananarivo, Madagascar; 4 Plate-forme Génomique, Institut Pasteur de Paris, Paris, France

## Abstract

**Background:**

Four of five *Plasmodium *species infecting humans are present in Madagascar. *Plasmodium vivax *remains the second most prevalent species, but is understudied. No data is available on its susceptibility to sulphadoxine-pyrimethamine, the drug recommended for intermittent preventive treatment during pregnancy. In this study, the prevalence of *P. vivax *infection and the polymorphisms in the *pvdhfr *and *pvdhps *genes were investigated. The correlation between these polymorphisms and clinical and parasitological responses was also investigated in *P. vivax*-infected patients.

**Methods:**

*Plasmodium vivax *clinical isolates were collected in eight sentinel sites from the four major epidemiological areas for malaria across Madagascar in 2006/2007. *Pvdhfr *and *pvdhps *genes were sequenced for polymorphism analysis. The therapeutic efficacy of SP in *P. vivax *infections was assessed in Tsiroanomandidy, in the foothill of the central highlands. An intention-to-treat analysis of treatment outcome was carried out.

**Results:**

A total of 159 *P. vivax *samples were sequenced in the *pvdhfr/pvdhps *genes. Mutant-types in *pvdhfr *gene were found in 71% of samples, and in *pvdhps *gene in 16% of samples. Six non-synonymous mutations were identified in *pvdhfr*, including two novel mutations at codons 21 and 130. For *pvdhps*, beside the known mutation at codon 383, a new one was found at codon 422. For the two genes, different combinations were ranged from wild-type to quadruple mutant-type. Among the 16 patients enrolled in the sulphadoxine-pyrimethamine clinical trial (28 days of follow-up) and after adjustment by genotyping, 3 (19%, 95% CI: 5%–43%) of them were classified as treatment failure and were *pvdhfr *58R/117N double mutant carriers with or without the *pvdhps *383G mutation.

**Conclusion:**

This study highlights (i) that genotyping in the *pvdhfr *and *pvdhps *genes remains a useful tool to monitor the emergence and the spread of *P. vivax *sulphadoxine-pyrimethamine resistant in order to improve the national antimalarial drug policy, (ii) the issue of using sulphadoxine-pyrimethamine as a monotherapy for intermittent preventive treatment of pregnant women or children.

## Background

*Plasmodium vivax *remains the second most common cause of malaria in the world, infecting more than 80 million people annually. It is the most geographically widespread malaria parasite and it is found throughout South and Central America, Asia, the Middle East and parts of Africa [1].

Malaria is endemic throughout Madagascar, except in highland regions above 1,500 m. *Plasmodium falciparum *is the dominant malaria species, but *P. vivax *and *Plasmodium malariae *have an increased prevalence in the foothills of the central highlands [2,3]. With over one million suspected cases reported in 2005, malaria remains one of the leading cause of morbidity and mortality in the country [4-6]. Although *P. vivax *causes less mortality than *P. falciparum*, it is responsible for significant morbidity and economic loss.

For the past 50 years, chloroquine was used as the first line treatment for malaria in Madagascar, with SP the second line treatment choice. In 2005, the National Malaria Control Programme (NMCP) decided to revise its treatment policy, replacing CQ by artemisinin-based combination therapy (AQ+AS, a combination of artesunate plus amodiaquine) and recommending SP for intermittent preventive treatment for pregnant women (IPTp) [7]. Some studies *in vivo *[8] and *in vitro *[9-11] have investigated the susceptibility of *P. falciparum *to this drug, but no data concerning the susceptibility of *P. vivax *to SP is available. Molecular and epidemiological studies have clearly shown that, as for *P. falciparum*, the major mechanism of resistance to SP involves specific point mutations in the *dhfr *(dihydrofolate reductase) and *dhps *(dihydropteroate synthase) genes of the parasite. In total, 20 non-synonymous mutations have already been described in the *pvdhfr *gene [12]. Some of these mutations (at codons 57, 58, 61, 117 and 173) are involved in resistance to pyrimethamine [12]. Five mutations have already been identified in the *pvdhps *gene, at codons 382, 383, 512, 553 and 585, corresponding to positions 436, 437, 540, 581 and 613 of the homologous gene in *P. falciparum*. No data is available about polymorphism in the *pvdhfr *and *pvdhps *genes of malaria parasites from Madagascar. Only one *pvdhfr *gene mutation, at codon 33, has been identified in some isolates (four of nine from Madagascar and the Comoro Islands), but this mutation has not been reported to be associated with clinical resistance or resistance *in vitro *[13,14].

Since, it is difficult to monitor the susceptibility of *P. vivax *to antimalarial drugs by *in vitro *tests [15], molecular markers of drug resistance are useful tools for mapping the current and changing pattern of SP-resistant *P. vivax *isolates. The aims of this study were: (i) to assess polymorphisms in the *pvdhfr *and *pvdhps *genes, using 159 *P. vivax *samples, and (ii) to correlate *pvdhfr/pvdhps *patterns with clinical and parasitological responses in *P. vivax*-infected patients.

## Methods

### Collection of clinical isolates of *P. vivax*

*Plasmodium vivax *clinical isolates were collected in 2006/2007 from individuals seeking treatment for malaria at government health facilities located in areas corresponding to the four epidemiological strata for malaria across Madagascar: Ejeda and Ihosy in the South (epidemic-prone area), Tsiroanomandidy and Moramanga/Saharevo in the foothills of the Central Highlands (low-level endemic area), Maevatanana and Miandrivazo in the West (seasonal and endemic area) and Farafangana and Andapa in the East (periannual endemic area). All patients with fever or a history of fever in the 48 h before their arrival at the health centre were screened with the rapid diagnostic test (RDT), which is based on the detection of *Plasmodium*-specific lactate dehydrogenase (pLDH) (OptiMAL-IT™, DiaMed AG^©^, Cressier sur Morat, Switzerland). Giemsa-stained thin and thick blood films were prepared for each patient with a positive RDT result. The various species of *Plasmodium *were identified and parasitaemia was assessed by a skilled microscopist. Once informed consent had been obtained from all adults and from at least one parent for minors, blood was collected on filter paper. Patients with positive microscopy results were promptly treated according to National Malaria Policy.

### SP efficacy

The therapeutic efficacy of the sulphadoxine-pyrimethamine was assessed in *P. vivax *infections in 2006 in Tsiroanomandidy. The clinical protocol was reviewed and approved by the National Ethics Committee of the Ministry of Health and Family Planning of Madagascar (N° 102-SANPF-2006). Patients with blood smear confirmed *P. vivax *infection were enrolled based on the inclusion criteria given in WHO guidelines (2001) [16]. Informed consent was obtained from all adults and from at least one parent for minors and patients were treated with the standard SP regimen (25 mg/kg sulphadoxine and 1.25 mg/kg pyrimethamine as a single dose on day 0) and followed for 28 days. Each patient was weighed and medical history (including previous antimalarial medication) was recorded. Clinical examination, including axillary temperature recording, staining of serial thick and thin films, was performed on days 0, 1, 2, 3, 7, 14, and 28. Giemsa-stained thick and thin films were read by a skilled microscopist. Asexual- and sexual-stage parasites were counted, and counts expressed per 200 white blood cells. Parasitaemia was calculated on the basis of a white blood cell count of 8,000/μl. Thick-film examinations were considered to be negative if no parasite was found in 100× high-power fields. Blood was blotted onto filter paper at various time-points during follow-up and stored at 4°C for DNA extraction. Haemoglobin concentration was measured on days 0 and 28 (HemoCue^©^, Anglholm, Sweden).

The primary end point was therapeutic response, based on parasitological and clinical cure by day 28, according to the 2001 WHO protocol [16]. Therapeutic response was classified as "treatment failure" (TF; clinical deterioration due to *P. vivax *illness requiring hospitalization, with parasitaemia and axillary temperature ≥ 37.5°C any time between days 3 and 28, or parasitaemia on any day between days 7 and 28, regardless of clinical conditions) or "adequate clinical and parasitological response" (ACPR; absence of parasitaemia on day 28 without the criteria for TF previously being met). Patients with treatment failure were treated with artesunate (4 mg/kg on days 0, 1, and 2) plus amodiaquine (10 mg/kg on days 0, 1, and 2); however, their response to repeat therapy was not assessed.

Molecular genotyping techniques were used to distinguish recrudescences from reinfections for patients with treatment failure after day 7. Briefly, blood samples collected on the day of enrolment, on day 1 and on the day of treatment failure were analysed by sequencing for polymorphisms in the genes for circumsporozoïte protein (*pvcsp*) [17] and merozoite surface protein-3 (*pvmsp3*) [18] and for six different microsatellite markers [19,20] (Table 1). Microsatellite PCR products were size-genotyped by using a standard-size Genescan 500 LIZ on an ABI Prism 3730XL. The genotyping patterns on the day of failure were compared with those at treatment initiation and on day 1. An outcome was defined as recrudescence if all alleles present at the time of failure were present at the time of treatment initiation or on day 1, and defined as a reinfection otherwise.

**Table 1:** Primer sequences of the microsatellite markers used for differentiating recrudescences from reinfections in paired samples from enrolled patients with treatment failure after day 7.

**Micro satellite markers**	**Motif**	**External PCR**	**Internal PCR**	**Label**
14.185^§^	AT	F: TGCAGATATGCTGTCGAAT	F: GCAGTTGTTGCAGATTGAGC	6FAM
		R: GGGAAAAACTTGGTCACAC	R: TAAGGCGTGCACGTTATCAT	
8.332^§^	AT	F: TGAAGCAATATAGCGATGAC	F: CCTCGATGGTGATGTGATGA	HEX
		R: CGGTGTAGTGTGGTACAATG	R: GTATAACATGGCACCCGACCT	
6.34^§^	AC	F: CCCAATTAAGTGCAAATCA	F: TGAGCGCTTTAAGCTTCTGC	6FAM
		R: CATGTAAAGAGGCACATGG	R: CAAAAATGAATCGTGGCACA	
2.21^§^	AC	F: GGCAGGAACGTAGAGGAG	F: CCATCTGCTCAAATCCGAAG	6FAM
		R: GGCTTGTTCATTTTGAGGTA	R: GGCTCCTCCCTGTCTCTGTAG	
AY391734^#^	CA	F: TACCCCAGCCTTATCTCTC	F: TTTTCCCTTCGGAAAAACG	6FAM
		R: AAATGCACAGACACTACGC	R: ACGACCATCACCTGCCATAG	
AY391740^#^	AC	F: ATTTGTGTATGCCTTGTGTT	F: GTTTACCAGGCCCAATTCAC	HEX
		R: GTGAGGGTGTCTATCCGTA	R: GTTCACACGGGCGTATACAT	

^§ ^microsatellites sequences described by Imwong et al, 2006 [20]

^# ^microsatellites sequences described by Leclerc et al, 2004 [19]

Primers sequences were designed by primer3 software [39]

F: forward primer

R: reverse primer

The secondary end points were (i) fever clearance time, defined as the time (days) from initiation of treatment to the end of fever (i.e., patient remaining afebrile, based on reported history, with an axillary temperature of <37.5°C on a given day of follow-up); (ii) parasite clearance time, defined as the time (days) from the initiation of treatment to parasite clearance (defined as the moment when parasite counts fell below the detection threshold for microscopy) and (iii) haematological recovery, defined as being cured on day 28, with a haemoglobin level greater than that on day 0.

### DNA extraction, *pvdhfr*/*pvdhps *amplication and sequencing

DNA was extracted from blood spots with Instagene^® ^Matrix resin (BioRad^©^, Marnes la Coquette, France), according to the manufacturer's instructions. Parasite species were confirmed by real-time PCR, using species-specific primers previously described by de Monbrison [21], with a protocol adapted for the RotorGene^® ^3000 thermocycler (Corbett Life Science^®^, Sydney, Australia).

*Plasmodium vivax *isolates were genotyped for the *pvdhfr *and *pvdhps *genes. DNA was amplified by semi-nested PCR for *pvdhfr *gene amplification or by nested PCR for *pvdhps*, using gene-specific primers [22,23]. PCR products were purified, using polyacrylamide P-100 Gel (Bio-Gel P-100, BioRad^®^, Marnes-la-Coquette, France), by 96-well plate filtration (Millipore^®^, St. Quentin en Yvelines, France). Sequencing reactions were performed using ABI PRISM BigDye Terminator cycle sequencing ready reactions kit and run on a 3730 xl Genetic Analyzer (Applied Biosystems, Courtaboeuf, France). Electrophoregrams were visualized and analysed with CEQ™ 2000 Genetic Analysis System software (Beckman Coulter™). Amino-acid sequences were compared with wild-type sequences (GenBank accession no. X98123 for *pvdhfr *and Genbank accession no. AY186730 for *pvdhps*), using BioEdit Sequence Alignment Editor software [24]. Parasites with mixed alleles (both wild-type and mutant alleles present) were considered to be mutant. A second PCR product was sequenced for confirmation if a new point mutation was observed.

### Nucleotide sequence accession number

The complete sequences of the alleles identified has been submitted to GenBank and assigned accession numbers EU168419 to EU168431 for *pvdhfr *and EU176992 to EU177003 for p*vdhps*.

### Statistical analysis

Data were input into EpiInfo 6.04^© ^software (Centers for Disease Control and Prevention, Atlanta, Georgia, United States), checked and analysed, using MedCalc^© ^software version 9.1.0.1 (MedCalc Software, Broekstraat 52, 9030 Mariakerke, Belgium) and Comprehensive meta-analysis software version 2 (Biostat, Englewood, NJ 07631, USA).

An intention-to-treat analysis of treatment outcome was carried out. Frequencies were compared using chi-squared tests, and continuous variables were compared using Student's t-tests or Mann-Whitney U-tests, as appropriate. All reported p-values are for two-tailed tests and were considered statistically significant if less than 0.05.

## Results

### Prevalence of *P. vivax *infections

**Figure 1:**
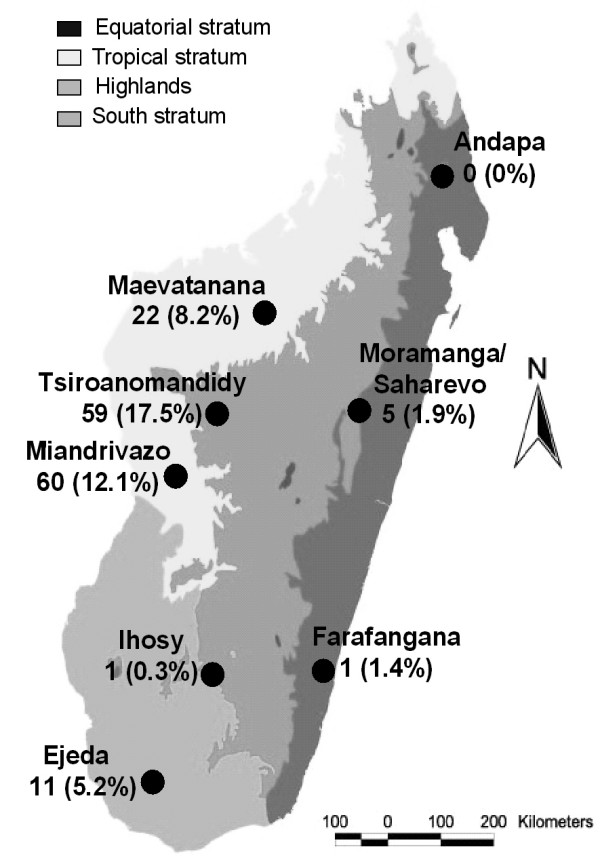
Map of Madagascar showing the distribution of *P. vivax *isolates from each study area.

From January to October 2006 and March to July 2007, 8,363 patients were screened by using RDT. Among these patients, the global prevalence of *P. vivax *infections was estimated at 6.3% (135 cases) of all malaria infections by microscopy. Considerable heterogeneity was observed between the different study areas and prevalence ranged from zero in Andapa to 17.5% in Tsiroanomandidy (Figure 1).

### Analysis of the *pvdhfr *and *pvdhps *genes

159 *P. vivax *samples, corresponding to the 135 cases completed with 24 collected in the same conditions, were sequenced on *pvdhfr/pvdhps *genes (Figure 1). A large proportion of these samples (71%, 113/159) had at least one mutation on the *pvdhfr *gene; this proportion ranged from 18% in Ejeda (2/11) to 100% in Ihosy and Farafangana (1/1). These samples displayed non-synonymous mutations at six residues, including four mutations known to be responsible for pyrimethamine resistance (P33L, C49R, S58R and S117N) and two previously unknown mutations (P21S and N130K). The most prevalent mutant-type alleles were S58R (58%) and S117N (63.5%), particularly for study sites on the western side of Madagascar. The mutant P33L allele, which has been reported to be specific to Madagascar, was found in only 6% of isolates and was not restricted to any particular area. The fourth mutation, C49R, was present in 3% of the isolates and was limited to Maevatanana. The two new non-synonymous mutations, P21S and N130K, were identified in two and 50 isolates, respectively, at only two sites (Miandrivazo and Tsiroanomandidy). An additional synonymous mutation was detected at amino-acid 15 (gca → gcg) in 69 samples (43%). No variation in the number of repeats was observed in the polymorphic region between nucleotides 262 and 309 [25].

Non-synonymous mutations were found at two residues of the *pvdhps *gene, in 25 of the 159 isolates: 24 samples (15%) displayed the A383G mutant allele and one sample (0.6%) the C422R mutant allele. No mutant alleles were found at southern sites and such alleles were surprisingly rare in Miandrivazo (3%), despite 83% of the isolates from this site having mutant alleles of the *pvdhfr *gene. Based on the *pvdhps *tandem repeat region located between amino-acid residues 603 to 658 of the reference sequence, nine different genotypes, including the reference genotype, were identified. All the A383G mutant-type isolates were from the same genotype. These changes and the corresponding positions of mutations in *pvdhfr *and *pvdhps *are shown in Table 2.

**Table 2:** Frequency distribution of mutant-type alleles for DHFR and DHPS domains in 159 Malagasy isolates collected from 7 sites in 2006–2007

			*P. vivax dhfr *polymorphism							*P. vivax dhps *polymorphism		
				
Sampling site		No. of isolates	No. of mutant isolates (%)	Amino-acid residue						No. of mutant isolates (%)	Amino-acid residue	
						
				**P21S**	P33L	C49R	S58R	S117N	**N130K**		A383G	**C422R**
South	Ejeda	11	2 (18)	-	1	-	1	1	-	0 (0)	0	0
	Ihosy	1	1 (100)	-	-	-	-	1	-	0 (0)	0	0
West	Miandrivazo	60	50 (83)	1	1	-	43	47	44	2 (3)	1	1
	Maevatanana	22	14 (64)	-	2	5	12	12	-	8 (36)	8	0
Central Highlands	Tdd	59	42 (71)	1	4	-	33	37	6	11 (19)	11	0
	Moramanga	5	3 (60)	-	1	-	2	2	-	3 (60)	3	0
East	Farafangana	1	1 (100)	-	-	-	1	1	-	1 (100)	1	0
Total		159	113 (71)	2 (1.3)	9 (5.7)	5 (3.1)	92 (57.9)	101 (63.5)	50 (31.4)	25 (16)	24 (15)	1 (0.6)

Residues displaying new mutations are indicated in bold typeface

Tdd: Tsiroanomandidy

In total, 15 different genotypes were identified, as presented in Table 3, including wild-type and single, double or triple mutant-types in for *pvdhfr *and the wild-type or single mutants for *pvdhps*. Only 27.7% of the 159 isolates sequenced were wild-type for both genes, and all but two of the parasites with mutant genotypes for *pvdhps *(98.7%) also had mutant genotypes for *pvdhfr*. Four single mutant allele genotypes were observed for *pvdhfr*, but none of these genotypes was ever associated with mutation in the *pvdhps *gene. In the double-mutant genotype, the 130K mutation was combined with 33L or 117N mutations in the *pvdhfr *gene, in the absence of mutation in the *pvdhps *gene. The *pvdhfr *58R/117N double mutant accounted for 25.8% of isolates, 10.1% of which also had the A383G mutation in the *pvdhps *gene. Finally, two triple-mutant *pvdhfr *genotypes were observed, with and without mutations in the *pvdhps *gene. The 49R/58R/117N genotype was found only in Maevatanana, whereas the 58R/117N/130K genotype was found mostly in Miandrivazo and Tsiroanomandidy.

**Table 3:** Frequency distribution of *pvdhfr*/*pvdhps *genotypes in 159 Malagasy isolates collected from 7 sites in 2006–2007

		**Sampling sites**							
			
***dhfr *alleles**	***dhps *alleles**	**South**		**West**		**Central Highlands**		**East**	**Total isolates (frequency %)**
						
		Ejeda	Ihosy	Maevatanana	Miandrivazo	Tdd	Moramanga	Farafangana	
***No mutation***									
Wild-type	Wild-type	9	-	7	10	17	1	-	44 (27.7)
	383G	-	-	1	-	-	1	-	2 (1.3)
***Single mutant***									
21S	Wild-type	-	-	-	1	1	-	-	2 (1.3)
33L	Wild-type	1	-	2	-	4	1	-	8 (5.0)
117N	Wild-type	-	1	-	3	3	-	-	7 (4.4)
130K	Wild-type	-	-	-	1	-	-	-	1 (0.6)
***Double mutant***									
33L + 130K	Wild-type	-	-	-	1	-	-	-	1 (0.6)
117N + 130K	Wild-type	-	-	-	1	1	-	-	2 (1.3)
58R + 117N	Wild-type	1	-	4	2	18	-	-	25 (15.7)
	383G	-	-	3	-	10	2	1	16 (10.1)
***Triple mutant***									
49R + 58R + 117N	Wild-type	-	-	1	-	-	-	-	1 (0.6)
	383G	-	-	4	-	-	-	-	4 (2.5)
	Wild-type	-	-	-	39	4	-	-	43 (27.0)
58R + 117N + 130K	422R	-	-	-	1	-	-	-	1 (0.6)
	383G	-	-	-	1	1	-	-	2 (1.3)

### Clinical and parasitological response to SP

Clinical and parasitological monitoring was complete up to day 28 for 15 of the 16 patients enrolled in the clinical study of SP efficacy. Nine of these 16 patients were male and seven were female (43.4%). They were aged from nine months to 42 years (median 7.5 years), and had a median weight of 16.5 kg (range 7.5 to 52.5). Fifteen patients (93.8%) had suffered fever during the 48 hours immediately preceding enrolment and one declared having taken antimalarial drugs (tetracycline). Neither microscopy nor real-time PCR showed mixed infections (*P. vivax *with other species). The geometric mean of asexual parasite count was 3,353 parasites/μL (range 500 to 21,500) at baseline. None of the patients had detectable gametocytes on microscopy at day 0 or during follow-up. Mean haemoglobin concentration was 7.5 g/dL on day 0, and the mean increase in haemoglobin concentration observed on day 28 was 1.3 g/dL. Eleven patients successfully cleared *P. vivax *parasitaemia after SP treatment, whereas four patients presented a reoccurrence of parasitaemia on days 14, 21 or 28. The final classification of the recrudescent patients after adjustment by genotyping is shown in Table 4. The treatment failure rate at day 28 by intention-to-treat analysis was also estimated to 25% (95% CI: 8–50) and after adjustment by genotyping to 19% (95% CI: 5–43). Mean fever clearance time was calculated as 1.2 ± 0.7 days and, parasite clearance time was calculated as 1.3 ± 0.9 days.

**Table 4:** Paired analysis of *pvcsp*, *pvmsp3 *and microsatellite markers sequences used for differentiating recrudescences from reinfections for the four patients with a reoccurrence of parasitemia (Tsiroanomandidy, Madagascar, 2006).

		**Allele sizes of microsatellites markers (bp)**						**Genotypes in**		
			
**Patient**	**Day**	**6.34**	**L34**	**2.21**	**8.332**	**14.185**	**L40**	***pvcsp gene***	***pvmsp3 gene***	**Final classification**
TDD062079	0	221/225	108	196	217	89/91	97	VK210	A	Recrudescence
	1	221/225	108	196	217	91	97	VK210	A	
	14	225	108	196	217	91	97	VK210	A	
TDD06viv12	0	219	108/110	196	222	na	97	VK210	na	Recrudescence
	1	219	108/110	196	222	na	97	VK210	na	
	28	219	110	196	222	na	97	VK210	na	
TDD06viv15	0	225	110	196	na	98	97	VK210	na	Reinfection or Relapse
	1	230	104	205	na	93	97	VK210	na	
	21	225	108	196	na	91	97	VK210	na	
TDD06viv20	0	230	108	198	na	91	97	VK210	A	Recrudescence
	1	230	108	198	na	91	97	VK210	A	
	14	230	108	198	na	91	97	VK210	A	

na: not available

Two infections (TDD062079 and TDD06viv12) were polyclonal as indicated by microsatellite markers analysis.

*pvcsp *sequences were classified as VK210 or VK247 types as described [17]; after DNA sequences alignment, no SNP was observed between day 0, 1 and the day of reoccurent parasitemia.

*pvmsp3 *sequences were classified into types A (1900 bp), B (1300 bp) or C (1100 bp) as described [40]; after DNA sequences alignment, one SNP was observed between TDD062079 at day 0 and the sequences obtained from both days 1 and 14. No differences were observed between TDD06viv20 sequences.

### *pvdhfr*/*pvdhps *genotypes and clinical response to SP

No significant differences were observed between recrudescent and non-recrudescent patients in terms of sex ratio, mean temperature at day 0, mean age, initial mean parasitaemia, haemoglobin concentration and mean number of mutations in *pvdhfr/pvdhps *genes (Table 5).

**Table 5:** Demographic, clinical and parasitological characteristics of baseline isolates from recrudescent and non-recrudescent patients (Tsiroanomandidy, Madagascar, 2006).

	Patients						
		
	Recrudescent			Non-Recrudescent			*P*
			
	No	Value	CI95%	No	Value	CI95%	
Patient charasteristics							
No of males/No of females	3	3/0	**-**	11	4/7	**-**	
Age (years), mean	3	5.3	0 – 15.6	11	14.7	5.3–24.0	NS
Temperature (°C), mean	3	38.8	35.8–41.9	11	38.0	37.6–38.4	NS
Hematology							
Hemoglobin concentration (g/dL), mean	3	6.8	5.5 – 8.1	11	7,5	5.1–9.9	NS
Isolates at the baseline							
Parasitemia (parasites/μL), geometric mean	3	3434	361 – 32627	11	3307	1603–6823	NS
Mean number of mutations in *dhfr/dhps *genes	3	2.3	1.6–3	11	1.4	0.6–2.2	NS

One patient presenting a *P. falciparum *infection and the one presenting a *P. vivax *reinfection (or relapse, as indicated by genotyping analysis) were excluded from the non-recrudescent patients group.

The *pvdhfr/pvdhps *genotype and therapeutic response of each isolate evaluated are listed in Table 6. Three of the four patients carrying the *pvdhfr/pvdhps *triple-mutant genotype (58R/117N, 383G) displayed ACPRs. The remaining patient was classified treatment failure at day 28. Considering the *pvdhfr *double mutant 58R/117N genotype, all 3 patients displaying treatment failure and 6 over the 11 displaying ACPRs carried it, providing an odd ratio for TF of 8.27 with a wide 95% CI of 0.35–197.6. The molecular/therapeutic outcome relationship can't be clearly depicted from these data.

**Table 6:** The *pvdhfr/pvdhps *genotypes and therapeutic responses of 15 patients from Tsiroanomandidy (Madagascar, 2006) treated with sulphadoxine-pyrimethamine for *P. vivax *infections

		Sequence polymorphism in					
Patient		*pvdhfr*			*Pvdhps*	No. of mutations in the 2 genes	Therapeutic response^b^
		
Trial no.	Age (Years)	33	58	117	383		
VIV7	2	P	S	S	A	0	ACPR
60446	2.5	P	S	S	A	0	ACPR
VIV14	7	**L**	S	S	A	1	ACPR
VIV18	13	P	S	S	A	0	ACPR
60756	42	P	S	S	A	0	ACPR
VIV3	12	P	**R**	**N**	**G**	3	ACPR
VIV8	34	P	S	**N**	A	1	ACPR
*VIV20*	*9*	P	**R**	**N**	A	2	TF
62298	5	P	**R**	**N**	A	2	ACPR
60250	6	P	**R**	**N**	A	2	ACPR
60149	8	P	**R**	**N**	**G**	3	ACPR
VIV4	30	P	**R**	**N**	**G**	3	ACPR
VIV12	6	P	**R**	**N**	**G**	3	TF
62079	0.8	P	**R**	**N**	A	2	TF
VIV15	2	P	**R**	**N**	A	2	Reinfection or relapse

## Discussion

This study highlights the real burden of *P. vivax *infections by updating prevalence data in eight sites throughout Madagascar. Despite the likely underestimation of the prevalence of *P. vivax *due to the use of the RDT for malaria screening [2], this study confirms, however, that *P. vivax *is the second prevalent species in malaria infections after *P. falciparum*. The highest prevalence of *Plasmodium vivax *infections were found in the western side of Madagascar, certainly because of the predominance of ethnic groups of Indo-Asian and Middle Eastern origin, who carry the Duffy antigen [26].

SP resistance and related single nucleotide polymorphisms (SNPs) in the *pvdhfr *and *pvdhps *genes were analysed in *P. vivax *samples from seven sites representative of the four major epidemiological strata for malaria. The two genes were screened by sequencing, to identify possible new mutations. The *P. vivax pvdhfr *gene is known to be highly diverse, supporting the use of such an approach [27]. A very high proportion of mutant-type isolates (72.2%) and diverse alleles were identified in these isolates, with 15 different mutant-type alleles observed if both *pvdhfr *and *pvdhps *gene mutations were taken into account. This is the first time that mutations at positions implicated in SP resistance have been described in *P. vivax *isolates from Madagascar.

Only two substitutions were identified in *pvdhps*, whereas six non-synonymous mutations, including four that have already been described, were found in *pvdhfr*. Mutations in these two genes do not play identical roles in the emergence of SP-resistance. Similar observations have been made for *P. falciparum *[28,29]. Mutations seem occurring first in *pfdhfr *gene, then after in *pfdhps *gene when most of the parasites in the population have double- or triple-mutant alleles of the *dhfr *gene [22].

Mutations in *pvdhfr*, including 58R and 117N, have been implicated in pyrimethamine resistance. The 58R allele was found in 58% of all *P. vivax *isolates, in combination with 117N, which was found alone or in combination with other mutations in 63.5% of all isolates. Studies *in vitro *have shown that the mutation of a single base, leading to the replacement of a serine by an asparagine residue at codon 117, increases the IC_50 _value of pyrimethamine by more than 80 times. The combination of this mutation with the replacement of a serine by an arginine residue at codon 58 generates an enzyme more than 400 times more resistant to pyrimethamine than the wild-type enzyme [30,31]. The 57L/58R/117N, triple mutant previously observed in Thailand [14] and associated with low levels of parasite clearance was not found. No parasites of the 57L/58R/61M/117T quadruple mutant type, associated with a high risk of SP treatment failure, were also not found.

Two new mutations were found at codons 21 and 130 in the *pvdhfr *gene. The mutation at codon 130 accounted for 31.4% of the isolates. This mutation, present mostly at Miandrivazo (67% of isolates), was strongly associated with the 58R/117N double mutant. The P33L substitution accounted for only 6% of the isolates. This mutation was previously found to be associated with isolates of Comorian or Malagasy origin [14,25]. One of the unique features of the *pvdhfr *gene of *P. vivax *is the presence of a tandem repeat between amino-acid residues 70 and 110. Size polymorphism has been reported in this region [22,32], but no variation was observed in this study.

It was impossible to assess the impact of the new N130K mutation based on clinical data, because only the 58R/117N double mutant was observed in isolates from the patients enrolled in the clinical trial of SP efficacy, including the isolate with the A383G mutation in *pvdhps*. The use of yeast constructs might facilitate interpretation of the role of this mutation in resistance [33].

Because of the absence of a genotyping consensus protocol for *P. vivax *to differentiate recrudescence from reinfection, the use of a combination of different genes such as *pvama1, pvmsp1, pvmsp3 *or microsatellite markers in paired analysis seems to be the safest available method. In this study, *pvcsp *and *pvmsp3 *genes sequencing and six different microsatellite markers were used on samples from day 0, day 1 and day of reoccurrence. The use of microsatellite markers seems to be useful as more polyclonal infections could be detected. Obvioulsy, the main limitation of this protocol was the well-recognized impossibility to prove that a *P. vivax *reoccurrence was a recrudescence, a relapse or a reinfection.

No significant association between *pvdhfr/pvdhps *polymorphisms and SP-treatment outcome was found, but all recrudescent patients were *pvdhfr *double-mutant carrier. This result strongly suggests that infection due to the *pvdhfr *58R/117N double mutant is necessary but not sufficient for SP treatment failure to occur. The treatment outcome is likely to be favourable if the parasite has a wild-type genotype, but in parasites with mutations, outcome depends on the alleles present at the *pvdhfr *and *pvdhps *loci and the individual response of the patient [12]. Currently, it is well known that beside parasite factors, host factors such as nutritional status, immune response and rates of drug metabolism are involved in determining treatment outcome.

Surprisingly, almost 72.3% of the tested *P. vivax *isolates had mutations in the *pvdhfr *and/or *pvdhps *genes, despite SP never having been recommended as a first-line treatment for malaria, but only as a second-line treatment from 1998 to 2005. ACT is now recommended for the treatment of uncomplicated malaria regardless of the causal *Plasmodium *species. Nevertheless, self-treatment remains frequent in Madagascar since unpublished study has shown that three quarters of all febrile patients attending government health facilities have already used chloroquine in 67.6% of cases, cotrimoxazole (sulphamethoxazol/trimethoprim) in 23.4% and SP in 9.4% of cases. Cotrimoxazole is the drug most widely used to treat diarrhoea as well as respiratory infections [34]. Asymptomatic *P. vivax *infections and treatment with trimethoprim are probably common, resulting in the exposure of parasites to this drug. Mutations in the *pvdhfr *and *pvdhps *genes may reflect overall antifolate drug pressure in Madagascar.

Previous studies have shown that *dhfr *mutant *P. falciparum *isolates were extremely rare [35], with only one case of infection with the 108N mutant reported in the south of Madagascar. Based the observations for *P. vivax *isolates, data for *P. falciparum *genotypes should be updated.

## Conclusion

SP has been recommended for intermittent preventive treatment during pregnancy since 2005 in Madagascar. The regional office of the WHO for Africa currently recommends IPTp with SP in countries with a parasitological failure rate of less than 50% [36]. With a frequency of TF estimated at 19% and a prevalence of up to 17.5% of all malaria cases, *P. vivax *infections remains a public health problem in Madagascar and there is no doubt that SP will not be completely effective in IPTp strategy [37,38]. The extensive use of SP will increase the drug selection pressure on the parasite and favour the spread of resistance. As a result, this study highlights (i) that genotyping in the *pvdhfr *gene remains a useful tool to monitor the emergence and the spread of *P. vivax *SP-resistant in order to improve the national antimalarial drug policy, (ii) the issue of using SP as a monotherapy for IPT of pregnant women or children.

## Authors' contributions

CBa performed laboratory work and wrote the manuscript. MT and CBo carried out sequencing and gave constructive advice. AR, LR and RR performed the field work. MJ performed laboratory work. SP helped with the writing of the manuscript and gave constructive advice. DM was involved in all stages of this study.
